# The relationship between learner engagement and teaching effectiveness: a novel assessment of student engagement in continuing medical education

**DOI:** 10.1186/s12909-020-02331-x

**Published:** 2020-11-04

**Authors:** Christopher R. Stephenson, Sara L. Bonnes, Adam P. Sawatsky, Lukas W. Richards, Cathy D. Schleck, Jayawant N. Mandrekar, Thomas J. Beckman, Christopher M. Wittich

**Affiliations:** 1 Division of General Internal Medicine, Mayo Clinic, 200 First St SW, Rochester, MN 55905 USA; 2 Division of Biomedical Statistics and Informatics, Mayo Clinic, Rochester, MN USA

**Keywords:** CME, Engagement, Validity, Teaching effectiveness

## Abstract

**Background:**

Continuing medical education (CME) often uses passive educational models including lectures. However, numerous studies have questioned the effectiveness of these less engaging educational strategies. Studies outside of CME suggest that engaged learning is associated with improved educational outcomes. However, measuring participants’ engagement can be challenging. We developed and determined the validity evidence for a novel instrument to assess learner engagement in CME.

**Methods:**

We conducted a cross-sectional validation study at a large, didactic-style CME conference. Content validity evidence was established through review of literature and previously published engagement scales and conceptual frameworks on engagement, along with an iterative process involving experts in the field, to develop an eight-item Learner Engagement Instrument (LEI). Response process validity was established by vetting LEI items on item clarity and perceived meaning prior to implementation, as well as using a well-developed online platform with clear instructions. Internal structure validity evidence was based on factor analysis and calculating internal consistency reliability. Relations to other variables validity evidence was determined by examining associations between LEI and previously validated CME Teaching Effectiveness (CMETE) instrument scores. Following each presentation, all participants were invited to complete the LEI and the CMETE.

**Results:**

51 out of 206 participants completed the LEI and CMETE (response rate 25%) Correlations between the LEI and the CMETE overall scores were strong (r = 0.80). Internal consistency reliability for the LEI was excellent (Cronbach’s alpha = 0.96). To support validity to internal structure, a factor analysis was performed and revealed a two dimensional instrument consisting of internal and external engagement domains. The internal consistency reliabilities were 0.96 for the internal engagement domain and 0.95 for the external engagement domain.

**Conclusion:**

Engagement, as measured by the LEI, is strongly related to teaching effectiveness. The LEI is supported by robust validity evidence including content, response process, internal structure, and relations to other variables. Given the relationship between learner engagement and teaching effectiveness, identifying more engaging and interactive methods for teaching in CME is recommended.

## Background

Continuing Medical Education (CME) courses are frequently used by physicians to maintain up-to-date professional competence and performance [1]. However, numerous studies have questioned the effectiveness of CME teaching methods [2–5]. Traditional CME courses use mostly passive educational models [6]. Although there is evidence that interactive teaching methods may be more effective than lectures, lectures remain the primary teaching modality in CME [1, 7, 8]. Ideally, education should be an active process where students are engaged to learn [9, 10].

Student engagement is a complex phenomenon that involves both physical and psychological constructs. It is defined as the amount of energy students physically and psychologically expend whereby they stay attentive, involved, and motivated to learn [11, 12]. Studies outside of CME demonstrate that more engaged students have higher course satisfaction and achievement of course learning objectives [13, 14]. Studies of college-level courses have reported that more engaged learners are also high achievers [15, 16]. In medical education, learner engagement in online CME courses is associated with improved patient outcomes including improved diabetic metrics [17]. Additionally, interactive teaching methods in CME are associated higher self-reported learning scores [7]. Despite studies suggesting that more engaged learners are more successful, research on learning engagement for in-person medical education, specifically continuing medical education, is limited.

Previously published conceptual frameworks divide engagement into multiple domains including behavioral engagement, psychological or emotional engagement and cognitive engagement [11, 12, 14, 18–24]. Additionally, these domains can be separated into in-class and out-of-class components [13, 14].

Behavioral engagement involves the physical actions of the learner [14, 18, 20]. This includes attendance, participation in class activities, and attentiveness [18, 20]. For example, a student who demonstrates high behavioral engagement would attend didactic sessions or workshops, avoid distractions, and actively participate by taking notes, using audience response methods, or participating in group discussions [19, 21, 23, 24].

Psychological engagement refers to the positive and negative emotions a learner experiences in response to the teacher, peers, and the education environment [13, 14, 18, 20]. A student with high emotional engagement would enjoy the education session and not focus on the passage of time. Low emotional engagement would include experiences and expressions of boredom, anger, anxiety or sadness [19, 20, 24].

Cognitive engagement involves an ability to engage in self-regulated learning and an appreciation for the value of learning. In cognitive engagement, students are motivated to learn, both in and out of the classroom [13, 14, 20]. Cognitive engagement also involves the perceived relevance of material to the learner’s experience. A student with high cognitive engagement would appreciate the applicability of material to future practice and would be motivated to learn more about the material, even outside of class [19, 24].

There are several engagement assessment instruments which have been published for secondary school and college-level students. However, these instruments have items and domains that are specific to these populations and that may not be relevant to practicing physicians attending CME [13, 14, 20, 25]. Furthermore, these instruments are cumbersome, having numerous items which would be impractical for assessing learner engagement following multiple lectures or workshops in CME populations. Since engaged learners are linked to higher academic achievement, measuring learner engagement in CME should be pursued [7, 13–17]. However, instruments to measure learner engagement in medical education, specifically CME, are lacking. Using Messick’s framework for validity of psychometric assessment [26], we aimed to develop a novel instrument to assess learner engagement in CME, supporting 1) content validity using previously published conceptual frameworks and prior instruments [13, 14, 18] and item development through an iterative process with experts in the field, 2) internal structure validity by performing factor analysis and calculations of internal consistency, and 3) relations to other variables by exploring associations with engagement scores, teaching methods, and burnout. Based on prior research, we hypothesized that learner engagement would consist of behavioral, psychological, and cognitive domains, be directly related to teaching effectiveness, and be inversely related to learner burnout [27].

## Methods

### Study design and participants

We conducted a cross-sectional study of attendees at the 27th Mayo Clinic Internal Medicine Board Review. This week-long course is a high-yield intensive program designed to assist learners with the American Board of Internal Medicine (ABIM) Initial and Recertification Examinations and to provide a relevant review for daily practice. Historically, most attendees are physicians who will be taking one of these examinations. The Mayo Clinic Internal Medicine Board Review offers 52.5 category 1 CME credits and consists of 57, 30–60 min podium presentations. This setting was selected due to its generalizability in CME.

Mayo Clinic faculty members are selected to present based on their topic-related expertise. Course content is determined by a planning committee of generalists, specialists, and course directors. Learning objectives are given to each presenter by the course directors. This study was deemed exempt by the Mayo Clinic Institutional Review Board.

### Learner engagement instrument (LEI) development

**Fig. 1:**
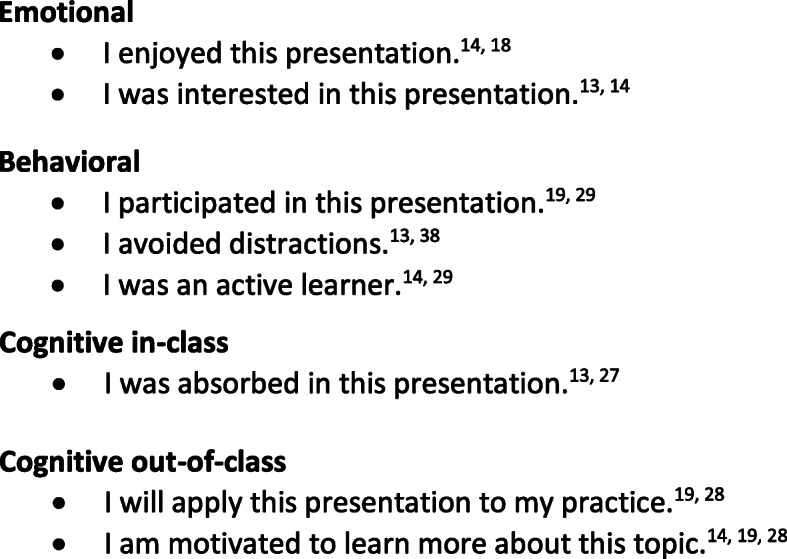
Learning Engagement Instrument. Emotional • I enjoyed this presentation [14, 18]. • I was interested in this presentation [13, 14]. Behavioral • I participated in this presentation [19, 29]. • I avoided distractions [13, 32]. • I was an active learner [14, 29]. Cognitive in-class • I was absorbed in this presentation [13, 27]. Cognitive out-of-class • I will apply this presentation to my practice [19, 28]. • I am motivated to learn more about this topic [14, 19, 28]

A Learner Engagement Instrument (LEI) was developed to evaluate learners’ engagement in CME. *To provide content validity, study authors and experts in the field [C.R.S., S.L.B., A.P.S., L.W.R., T.J.B., C.M.W.] reviewed conceptual frameworks on engagement and previously published engagement instruments* [13, 14, 18–20, 25, 28–31]. *Items were generated from both the conceptual frameworks and from the previously published instruments. Items were then revised through an iterative process until a consensus was achieved among the study authors.* The conceptual framework included three domains of engagement: emotional engagement, behavioral engagement, and cognitive engagement. Cognitive engagement was divided into cognitive in-class and cognitive out-of-class, as supported by previous literature [13]. An eight-item instrument was created and blueprinted to the three domains as shown in Fig. 1. The eight items were: 1) I enjoyed this presentation [14, 18], 2) I was interested in this presentation [13, 14], 3) I participated in this presentation [19, 29], 4) I avoided distractions [13, 20] 5) I was an active learner [14, 29], 6) I was absorbed in this presentation [13, 27], 7) I will apply this presentation to my practice [19, 28], 8) I am motivated to learn more about this topic [14, 19, 28].

### CME teaching effectiveness (CMETE) instrument

All presentations were evaluated using a previously validated CME teaching effectiveness (CMETE) instrument [33]. The CMETE instrument is a unidimensional survey containing eight items on a five point ordinal scale (1 = strongly disagree, 2 = disagree, 3 = neutral, 4 = agree, 5 = strongly agree). The instrument scores previously demonstrated content, internal structure and relations to other variable validity evidence [33]. Learners completed both the CMETE and LEI after each lecture presentation. To prevent survey fatigue, two authors (C.P.W. and T.J.B.) from the original CMETE validation study reviewed the CMETE instrument to decrease the number of items. Through an iterative process, the CMETE was decreased from eight items to four items. The items with the highest interclass-correlation from the initial CMETE validation study were retained [33]. The final four items were: 1) Speaker presented information in a clear and organized manner, 2) Examples or cases were given that facilitated my understanding, 3) The slides added to the effectiveness of the presentations, and 4) Speaker included opportunities to learn interactively.

### Data collection

At the CME course, all attendees were invited to participate in the study. Attendees were reminded to download the conference app that collected: 1) demographic questions, 2) the CME Teaching Effectiveness (CMETE) Instrument for each presentation, and 3) The Learner Engagement Instrument (LEI) for each presentation. Previous research supports the response process validity evidence for the app used in this study [33]. All information collected remained anonymous. Participant demographic characteristics include age (years), gender (male, female), practice type (academic, group, solo, government or military, retired), specialty (family medicine, internal medicine, etc.), time spent providing patient care per week (hours), and years in clinical practice (years).

Burnout was assessed using a previously validated 2-item instrument [34]. Burnout was selected as a measure since previous research has demonstrated an inverse relationship to engagement [27]. Participants recorded their CMETE instrument scores and LEI responses within the app, which anonymously linked to their demographic data.

Presenter variables included: age (years), academic rank, medical student teaching within the last year (yes or no), resident teaching within the last year (yes or no), fellowship teaching within the last year (yes or no), CME teaching within the last year (yes, no), and provision of direct patient care within the last year (yes or no).

Presentation characteristics included number of slides, summary slides, time of day (morning or afternoon), day of conference, and use of a pearls format. As outlined in prior research, slides which included titles of “pearls/clinical pearls” were counted as using the pearls format [35]. A presentation was considered to have a summary slide if a slide listed “Key points”, “Outline”, “Take home message”, “Learning points”, “Some sort of summarized descriptions of the objectives”, or “Conclusions.”

### Statistical analysis

The data were entered and processed by the Mayo Clinic Survey Research Center. Categorical variables were presented percentages and numbers. To build validity to internal structure, an exploratory factor analysis was performed. The minimal proportion criteria were used for factor extracting. Items with factor loadings ≥0.50 were retained. Internal consistency reliability for items comprising each factor was calculated with a Cronbach alpha of > 0.7 as acceptable [36].

To account for the clustering of multiple ratings by students completing more than 1 evaluation, we generated an adjusted correlation matrix using mixed model approach. This adjusted correlation matrix was then used to perform confirmatory factor analysis with orthogonal rotation. In addition, for a sensitivity analysis, we also performed factor analysis using an unadjusted correlation matrix (Table 2).

For relations to other variables validity, associations between LEI scores and participant, presenter and presentation characteristics were determined using the Wilcoxon statistic. The level of statistical significance was set at an alpha of 0.05. Statistical analyses were conducted using SAS version 9.4 (SAS Institute Inc., Cary, NC).

## Results

Demographics are reported in Table 1. A total of 51 out of 206 participants completed the LEI, CMETE and demographic survey (response rate 25%). However, since participants completed surveys on up to 57 lectures, 2486 LEI and CMETE surveys were submitted out of a total of 2907 possible surveys for the 51 participants (86% of possible presentations). Of the 51 participants, 30 (59%) were male and 40 (78%) were internal medicine physicians. Over half of respondents (57%) were over the age of 50. The majority of participants were from the Midwestern United States (*n* = 35, 69%).

**Table 1:** Demographics of Participants

	Total (*N* = 51)
**Practice Setting**	
Academic	10 (19.6%)
Govt/Military	3 (5.9%)
Group	30 (58.8%)
Solo	3 (5.9%)
other	5 (9.8%)
**Specialty**	
IM	40 (78.4%)
FM	2 (3.9%)
Medical	8 (15.7%)
Non-Medical	1 (2.0%)
**Practice Location**	
Northeast	2 (3.9%)
Southeast	9 (17.6%)
Midwest	35 (68.6%)
Southwest	3 (5.9%)
West	2 (3.9%)
**Reason for Enrolling**	
New board certification	15 (29.4%)
Renewal of certification	19 (37.3%)
Not passing prior certification	1 (2.0%)
General knowledge	16 (31.4%)

To support validity to internal structure, an exploratory factor analysis of the LEI was performed as shown in Table 2. A sensitivity analysis accounting for the clustering of multiple ratings by students completing multiple evaluations showed no differences between the factor analyses using the adjusted or unadjusted correlation matrixes. Items with a factor loading of 0.5 were retained with no items needing to be removed. Two factors were identified. One factor, which consisted of 4 items, included the domains of emotional engagement and cognitive out-of-class. We termed this new combined domain, “internal engagement.” Internal engagement reflected items which were internal to the learner, such as their enjoyment of the topic and motivation for self-directed learning and applicability of the material. The other factor comprised of the remaining 4 items and including the domains of behavioral engagement and cognitive in-class. We termed this new combined domain, “external engagement.” External engagement consisted of items external to the learner, which could be observed, such as participation during the course and being an active, absorbed learner. The internal consistency reliabilities (Cronbach alpha) were 0.96 for the internal engagement domain and 0.95 for the external engagement domain. Internal consistency was 0.96 for all 8 items overall. The abbreviated 4-item CMETE instrument’s internal consistent was 0.97 which is similar to previously published studies on this instrument’s performance [33].

**Table 2:** Exploratory Factor Analysis of Learner Engagement Instrument (Unadjusted)

Questions	Factor 1	Factor 2
Enjoyed presentation	0.7013	0.4627
Interested presentation	0.7471	0.4983
Participated presentation	0.3751	0.6803
Avoided distractions	0.4372	0.7046
Active listener	0.4940	0.7487
Absorbed presentation	0.4710	0.7223
Apply presentation practice	0.7357	0.4172
Motivated learn more	0.7499	0.4420
**Exploratory Factor Analysis of Learner Engagement Instrument (Adjusted for Correlation)**		
**Questions**	**Factor 1**	**Factor 2**
Enjoyed presentation	0.7120	0.4467
Interested presentation	0.7598	0.4761
Participated presentation	0.3619	0.6996
Avoided distractions	0.4364	0.7106
Active listener	0.5102	0.7334
Absorbed presentation	0.4533	0.7372
Apply presentation practice	0.7377	0.4115
Motivated learn more	0.7546	0.4337

To support relations to other variables validity, the LEI was compared to the CMETE, presentation characteristics, and burnout. Mean scores for the LEI and CMETE are reported in Table 3. The LEI was highly correlated to the CMETE (R = 0.80). Internal engagement correlated with teaching effectiveness (R = 0.80) stronger than external engagement (R = 0.73) There were no differences in the LEI or CMETE scores based on demographics including age, sex, practice location, specialty or reason for enrolling in the course.

**Table 3:** Results of Learner Engagement Instrument and CME Teaching Effectiveness Instrument

	Total (***N*** = 51)
**Learner Engagement instrument (LEI)**	
Mean (SD)	35.6 (7.3)
Median	38.1
Q1, Q3	34.0, 39.8
Range	(0.0–40.0)
Internal consistency	0.96
**Factor 1 of LEI (Internal Engagement)**	
Mean (SD)	17.9 (3.7)
Median	19.4
Q1, Q3	17.4, 19.8
Range	(0.0–20.0)
Internal consistency	0.96
**Factor 2 of LEI (External Engagement)**	
Mean (SD)	17.7 (3.7)
Median	19.0
Q1, Q3	16.4, 19.9
Range	(0.0–20.0)
Internal consistency	0.95
**CME Teaching Effectiveness Instrument**	
Mean (SD)	18.1 (3.8)
Median	19.4
Q1, Q3	17.6, 19.9
Range	(0.0–20.0)
Internal consistency	0.97

Presentations on the first day of conference had lower mean LEI scores compared to later days (− 0.21 on a 5 point scale, *p* < 0.001). Presentations on the first day of conference also had lower CMETE scores (− 0.19 on a 5 point scale, *p* < 0.001). Otherwise, presentation characteristics, including use of humor, number of slides, summary slides or use of pearl format and time of day did not influence CMETE or LEI scores. Learners with burnout tended to have higher learning engagement scores, but the results were not significant (− 0.79 on a 5 point scale, *p* = 0.41).

## Discussion

The Learner Engagement Instrument (LEI) is an innovative method for measuring engagement in the CME population. The LEI demonstrated strong internal structure validity evidence including a two-factor model that measures engagement in both psychological and behavioral domains. We also identified relations to other variables evidence in terms of a strong relationship between learner engagement and teaching effectiveness, which suggests that effective teachers are more likely to engage their learners.

The LEI is supported by validity evidence according to Messick’s framework for assessment [26]. Regarding content validity evidence, the LEI underwent a rigorous design process that incorporated previously published literature and conceptual frameworks on engagement, along with input from experts and an iterative process of item development. Additionally, to preserve the integrity of the data and support validity to response process, we used a well-developed online platform to collect information with clear instructions was utilized. Additionally, prior to implementation, all LEI items were pilot tested on physicians outside of the study with feedback given on clarity and the perceived meaning of the items. All items were entered and analyzed using a dedicated survey research center. Finally, internal structure validity evidence was supported by factor analysis demonstrating a multidimensional assessment of learner engagement in CME, as well as excellent internal consistency reliability.

Furthermore, relation to other variables validity was supported by substantial correlations between overall LEI and CMETE scores. In summary, effective teachers had more engaged learners. Since the CMETE included items such as speaker clarity, use of case-based presentations, organized slides, and opportunities to learner interactively, faculty should be encouraged to employ these strategies routinely in their teaching sessions. Presentations on the first day of the course had slightly lower LEI scores than presentations on later days. However, this may be related to teaching effectiveness since these presentations also had lower CMETE scores.

We observed a trend towards increased burnout and more engagement rather than less engagement as was previously hypothesized. However, it’s possible that participants with higher burnout tend to exhibit higher focus, or perhaps participants with higher burnout have reasons to be more concerned about their future performance on certification examinations. Furthermore, the high-stakes nature of a board review course may contribute to increased burnout. More study is needed to identify if engagement and burnout are related in a CME population. Studying engagement and burnout in a lower stakes CME environment should also be considered.

**Fig. 2:**
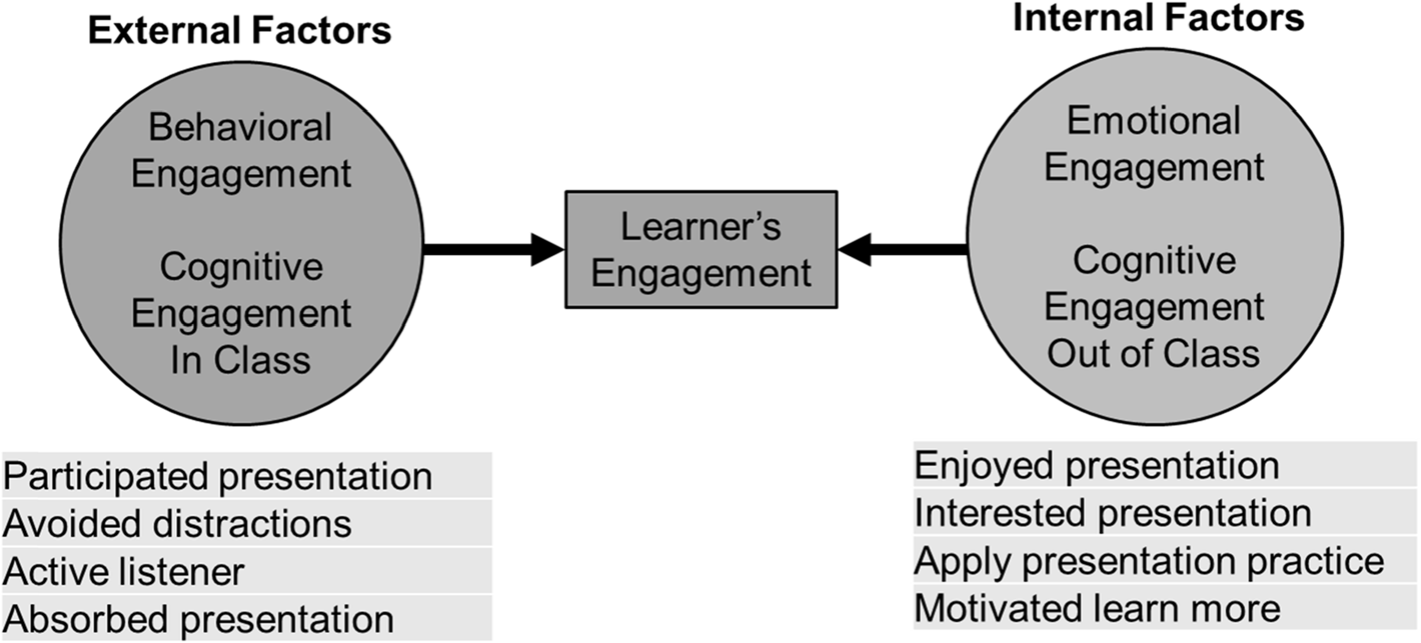
Revised Conceptual Framework on Engagement

Though we expected three domains, our exploratory factor analysis of the LEI revealed a two-dimensional instrument. A two-domain conceptual framework for engagement is also supported in the literature [12]. The revised framework for engagement based on our factor analysis is shown in Fig. 2. The items of emotional engagement and cognitive out-of-class engagement loaded together in the factor analysis. These domains focused on items internal to the learner, which cannot be seen by an outside observer. This includes a learner’s enjoyment and interest in the topic as well as the learner’s motivation to learn and apply the topic in their practice [19, 23, 24].Given that these items were internal preferences or motivations, we termed this new domain, “Internal Engagement.”

Additionally, behavioral engagement and cognitive in-class engagement loaded together in the factor analysis. Both these domains focused on the external actions of the learner while in class. This includes participating, avoiding distractions, listening and being absorbed in the presentation [19, 23, 24]. Given that these items were in-class actions which could be observed by an external observer, we termed this engagement, “External Engagement.”

The domains of internal and external engagement parallel concepts in self-determination theory. Self-determination theory describes how humans are motivated to learn and includes concepts of amotivation, extrinsic motivation and intrinsic motivation [37]. Intrinsic motivation is internal and includes the learner’s personal interest and enjoyment of the material [38]. This is similar to our identified domain of internal engagement. A learner who has high internal engagement may find a topic enjoyable and align with their personal interests. Conversely, extrinsic motivation describes external pressures that influence a learner’s motivation to learn. This could include career goals, rewards, or societal demands [38]. A learner who has a high external engagement may be cognizant of the social pressures to avoid distractions and be an active participant, consequently also having high external engagement in the presentation. More research is needed to further explore the overlap between these theories on engagement and motivation to learn.

There are several reasons to explain why a third domain wasn’t identified in our factor analysis. First, previously published engagement inventories at the high school and college-level include more items for each domain [13, 14, 25]. Given that the LEI had only eight items, there may be too few items to discriminate between various domains of engagement. Second, as outlined in previous conceptual frameworks, there may only be two domains of engagement, a psychological-based domain and behavioral-based domain, with cognitive engagement existing in an overlap between the two. Third, CME attendees may be inherently different from college-level students and the domain of cognitive engagement may not be relevant to this population. Lastly, as noted above, our framework for engagement may overlap with the concepts of motivations to learn where internal and external engagement relate to intrinsic and extrinsic motivation to learn.

Our study has several strengths. As previously noted, the LEI is supported by strong validity evidence using Messick’s framework to design the instrument and underwent a vigorous design process [26]. Our sample was collected in a large, didactic-style CME course, which is representative of most types of CME. Last, although our response rate was modest, over 2500 LEI and CMETE surveys were answered. There are also several limitations. As noted above, our response rate was low to modest, which likely introduces selection bias. It is possible that only the most engaged learners completed the survey, biasing our results. This could artificially inflate learner engagement scores and skew our results. Furthermore, we were unable to collect comparative information on nonresponders. Studying the engagement of these initial nonresponders could be a potential area for future research. Additionally, engagement isn’t dichotomous and we didn’t identify a score cutoff differentiating between highly engaging presentations and non-engaging ones.

This smaller sample size may have also contributed to a type II error for burnout if our sample size was not adequate to detect a relationship between engagement and burnout. Future research on the relationship between burnout and engagement should be sought with larger sample sizes. Furthermore, additional study on engagement and relationship to teaching effectiveness in other populations, such as undergraduate and graduate medical education should be pursued.

## Conclusion

Engagement is an important construct for learner retention, but measuring engagement can be challenging. Overall, the LEI demonstrated strong validity evidence and showed that more effective teachers, as assessed by students’ evaluations, were associated with higher student engagement. Given the relationship between learner engagement and teaching effectiveness, identifying more engaging and interactive methods for teaching in CME is recommended.

## Data Availability

Data for this study is available at the following hyperlink: https://www.dropbox.com/s/wrkjc29g1gv41cd/2018%20Engagement%20Dataset.xlsx?dl=0

## Abbreviations

CME: Continuing Medical Education
ABIM: American Board of Internal Medicine
LEI: Learner Engagement Instrument
CMETE: Continuing Medical Education Teaching Effectiveness
